# Genome sequences published outside of *Standards in Genomic Sciences,* May-June 2012

**DOI:** 10.4056/sigs.3126494

**Published:** 2012-07-20

**Authors:** Oranmiyan W. Nelson, George M. Garrity

**Affiliations:** 1 Editorial Office, Standards in Genomic Sciences and Department of Microbiology, Michigan State University, East Lansing, MI, USA

## Abstract

The purpose of this table is to provide the community with a citable record of publications of ongoing genome sequencing projects that have led to a publication in the scientific literature. While our goal is to make the list complete, there is no guarantee that we may have omitted one or more publications appearing in this time frame. Readers and authors who wish to have publications added to subsequent versions of this list are invited to provide the bibliographic data for such references to the SIGS editorial office.

**Domain Archaea**
Phylum *Euryarchaeota*
*Thermococcus zilligii* AN1, sequence accession AJLF00000000 [1]

**Domain Bacteria**
Phylum Deinococcus-Thermus
*Thermus sp.* Strain RL, sequence accession AIJQ00000000 [2]
Phylum *Proteobacteria*
*Azospira** suillum* Strain PS, sequence accession CP003153 [3]
*Bradyrhizobium elkanii* 587, sequence accession AJJK00000000 [4]
*Burkholderia sp.* strain SJ98, sequence accession AJHK00000000 [5]
*Corallococcus coralloides* DSM 2259, sequence accession CP003389 [6]
*Diplorickettsia massiliensis* strain 20B, sequence accession AJGC00000000 [7]
*Escherichia coli* DECA collection, AIEV00000000 through AIEZ00000000, AIFA00000000 through AIFZ00000000, AIGA00000000 through AIGZ00000000, AIHA00000000 through AIHS00000000 [8]
*Escherichia coli* O157:H7 strain EDL933, sequence accession NC_002655.2 [9]
*Escherichia coli* J53, sequence accession AICK00000000 [10]
*Escherichia coli* Strain NCCP15657, sequence accession AJLU00000000 [11]
*Escherichia coli* NCCP15647, sequence accession AJMB00000000 [12]
*Escherichia coli* NCCP15658, sequence accession AJMD00000000 [13]
*Francisella philomiragia* ATCC 2501, sequence accession NC_010336.1 [14]
Gammaproteobacterial BDW918, sequence accession AJMK00000000 [15]
*Glaciecola punicea* ACAM 611^T^, sequence accession BAET00000000 [16]
*Halomonas sp.* Strain KM-1, sequence accession BAEU01000001 through BAEU01000173 [9]*Vibrio sp.* Strain EJY3, sequence accession CP003241 and CP003242 [17]
*Helicobacter cinaedi* Strain PAGU611, sequence accession AP012344 and AP012345 [18]
*Helicobacter pylori* hpEurope Strain N6, sequence accession CAHX01000001 through CAHX01000054 [19]
*Kingella kingae* septic arthritis isolate PYKK081, sequence accession AJGB00000000 [20]
*Klebsiella oxytoca* 11492-1, sequence accession AIEM01000000 [21]
*Klebsiella pneumoniae* strain KCTC 2242, sequence accession CP002910 and CP002911 [22]
*Klebsiella pneumoniae* Strain LCT-KP214, sequence accession AJHE00000000 [23]
*Marinobacter hydrocarbonoclasticus* SP17, sequence accession FO203363 [24]
*Pantoea sp.* Strain Sc 1, sequence accession AJFP00000000 [25]
*Pasteurella multocida* HN06, sequence accession CP003313 (chromosome) and CP003314 (plasmid) [26]
*Phaeospirillum molischianum* DSM120, sequence accession CAHP01000001 through CAHP01000061 [27]
*Providencia stuartii* Clinical Isolate MRSN 2154, sequence accession CP003488 [28]
*Pseudoalteromonas arctica* A 37-1-2, sequence accession AHBY00000000 [29]
*Pseudoalteromonas citrea* NCIMB 1889, sequence accession AHBZ00000000 [29]
*Pseudoalteromonas flavipulchra* JG1, sequence accession AJMP00000000 [29]
*Pseudoalteromonas marina* mano4, sequence accession AHCB00000000 [29]
*Pseudoalteromonas piscicida* JCM 20779, sequence accession AHCC00000000 [29]
*Pseudoalteromonas spongiae* UST010723-006, sequence accession AHCE00000000 [29]
*Pseudoalteromonas undina* NCIMB 2128, sequence accession AHCF00000000 [29]
*Pseudoalteromonas haloplanktis* ATCC 14393, sequence accession AHCA00000000 [29]
*Pseudomonas aeruginosa* Strain ATCC 27853, sequence accession AJKG00000000 [30]
*Pseudomonas fragi* Strains A22, sequence accession AHZY01000001 through AHZY01000114 [31]
*Pseudomonas fragi* Strains B25, sequence accession AHZX01000001 through AHZX01000227 [31]
*Pseudomonas fuscovaginae*, sequence accession AIEU00000000 [32]
*Pseudomonas geniculata* N1, sequence accession AJLO00000000 [33]
*Pseudomonas mandelii* Strain JR-1, sequence accession AJFM00000000.1 [34]
Pseudomonas sp. strain R62, sequence accession AHZM00000000 [35]
Pseudomonas sp. strain R81, sequence accession AHZN00000000 [35]
*Pseudomonas syringae* pathovar syringae strain FF5, sequence accession ACXZ00000000 [36]
*Rahnella aquatilis* CIP 78.65, sequence accession CP003244 (chromosome), CP003245 (plasmid pRahaq201), CP003246 (plasmid pRahaq202), CP003247 (plasmid pRahaq203) [37]
*Rickettsia conorii* subsp. indica, sequence accession AJHC00000000 [38]
*Rubrivivax gelatinosus* CBS, sequence accession AJFF00000000 [39]
*Rubrivivax gelatinosus* IL144, sequence accession AP012320 [40]
*Salmonella enterica* serovar Heidelberg strain 41563, sequence accession AJGX00000000 [41]
*Salmonella enterica* serovar Heidelberg strain 41565, sequence accession AJHA00000000 [41]
*Salmonella enterica* serovar Heidelberg strain 41566, sequence accession AJGZ00000000 [41]
*Salmonella enterica* serovar Heidelberg strain 41573, sequence accession AJGY00000000 [41]
*Salmonella enterica* serovar Heidelberg strain 41579, sequence accession AJGW00000000 [41]
Salmonella enterica subsp. enterica Serotype Heidelberg Strain B182, sequence accession CP003416 (chromosome) CP003417 (plasmid) [41]
*Serratia sp.* Strain M24T3, sequence accession AJHJ00000000 [42]
*Shigella flexneri* serotype 5a strain M90^T^ Sm, sequence accession AGNM00000000 [43]
*Sphingomonas sp.* strain PAMC 26621, sequence accession AIDW00000000 [44]
*Sphingomonas sp.*, sequence accession AHHA00000000 [45]
*Sphingomonas wittichii* DP58, sequence accession AHKO00000000 [46]
*Stenotrophomonas maltophilia* D457, sequence accession HE798556 [47]
*Vibrio campbellii* PEL22A, sequence accession AHYY00000000 [48]
*Vibrio cholerae* O1, sequence accession CP003330 and CP003331 [49]
*Vibrio cholerae* strain IEC224, sequence accession CP003330 (chromosome I), CP003331 (chromosome II) [50]
*Xanthomonas citri* pv. mangiferaeindicae strain LMG 941, sequence accession CAHO01000001 through CAHO01000195 [51]
Phylum *Firmicutes*
*Bacillus cereus* strain LCT-BC244, sequence accession AJGQ00000000 [52]
*Bacillus cereus* bacteriophage PBC1, sequence accession JQ619704 [52]
*Bacillus licheniformis* WX-02, sequence accession AHIF00000000 [53]
*Bacillus methanolicus* MGA3, sequence accession ADWW00000000 [54]
*Bacillus methanolicus* PB1, sequence accession AFEU00000000 [55]
*Bacillus sp.* strain JS, sequence accession CP003492 [56]
*Bacillus sp.* strain 5B6, sequence accession AJST00000000 [57]
*Bifidobacterium breve* CECT 7263, sequence accession AFVV00000000 [58]
*Clostridium arbusti* SL206^T^, sequence accession BAEV00000000 [59]
*Clostridium thermocellum* Wild-Type Strain YS, sequence accession AJGT00000000 [60]
*Clostridium thermocellum* AD2, sequence accession AJGS00000000 [61]
*Enterococcus faecium* strain LCT-EF90, sequence accession AJKH00000000 [62]
*Lactobacillus salivarius* SMXD51, sequence accession AICL00000000 [63]
*Lactobacillus vini* Strains LMG 23202T, sequence accession AHYZ00000000 [64]
*Lactobacillus vini* Strains JP7.8.9, sequence accession AHZA00000000 [65]
*Listeria monocytogenes* 07PF0776, sequence accession CP003414 [66]
*Melissococcus plutonius* DAT561, sequence accession AP012282 and AP012283 [67]
*Paenibacillus mucilaginosus* 3016, sequence accession CP003235 [68]
*Rahnella aquatilis* CIP 78.65, sequence accession [69]
*Sporolactobacillus vineae* SL153^T^, sequence accession BAEY01000001 through BAEY01000092 [70]
*Staphylococcus aureus* 118 (ST772), sequence accession AJGE00000000 [71]
*Staphylococcus aureus* C160, sequence accession ACUV00000000 [72]
*Staphylococcus aureus* C427, sequence accession ACSQ00000000 [72]
Phylum Actinobacteria
*Blastococcus saxobsidens* DD2, sequence accession FO117623 [73]
*Corynebacterium diphtheriae* bv. mitis NCTC 3529, sequence accession AJGI00000000 [74]
*Corynebacterium pseudotuberculosis* strain Cp267, sequence accession CP003407 [75]
*Microbacterium laevaniformans* Strain OR221, sequence accession AJGR00000000 [76]
Mycobacterium abscessus subsp. bolletii BD^T^, sequence accession AHAS00000000 [77]
*Mycobacterium abscessus* strain M93, sequence accession AJGF00000000 [78]
*Mycobacterium intracellulare* clinical strain MOTT-02, sequence accession CP003323 [79]
*Mycobacterium intracellulare* clinical strain MOTT-64, sequence accession CP003324 [80]
*Mycobacterium intracellulare* Strain ATCC 13950^T^, sequence accession CP003322 [81]
*Mycobacterium phlei* Type Strain RIVM601174, sequence accession AJFJ00000000 [82]
*Mycobacterium tuberculosis* Erdman, sequence accession AP012340 [83]
*Mycobacterium xenopi* Type Strain RIVM700367, sequence accession AJFI00000000 [84]
*Nocardia brasiliensis* HUJEG-1, sequence accession AIHV00000000 [85]
*Propionibacterium acnes* strain PRP-38, sequence accession AIJP00000000 [86]
Strain IMCC13023, sequence accession AJKR00000000 [87]
*Rhodococcus sp.* Strain P14, sequence accession AJFC00000000 [88]
*Rhodococcus imtechensis* RKJ300, sequence accession AJJH00000000 [89]
*Streptomyces somaliensis* DSM 40738, sequence accession AJJM00000000 [90]
*Streptococcus pneumoniae* Strain ST556, sequence accession CP003357 [91]
*Streptococcus mutans* Strain LJ23, sequence accession AP012336 [92]
Phylum *Planctomycetes*
*Schlesneria paludicola*, sequence accession AHZR00000000 [93]
*Singulisphaera acidiphila*, sequence accession AHZQ00000000 [93]
*Zavarzinella formosa*, sequence accession AIAB00000000 [93]
Phylum *Spirochaetes*
*Borrelia crocidurae*, sequence accession CP003426 (chromosome), CP003427 through CP003465 (plasmids) [94]
Phylum Bacteroidetes
*Aquimarina agarilytica* ZC1, sequence accession AHHE00000000 [95]
*Fibrella aestuarina* BUZ 2^T^, sequence accession HE796683 (chromosome) and HE796684 (plasmid) [96]
*Flavobacterium columnare* ATCC 49512, sequence accession CP003222 [97]
*Flavobacterium indicum* GPSTA100-9T, sequence accession HE774682 [98]
Gillisia* sp.* strain CBA3202, sequence accession AJLT00000000 [99]
*Imtechella halotolerans* K1^T^, sequence accession AJJU00000000 [100]
Myroides *injenensis* M09-0166^T^, sequence accession [101]
*Marinilabilia salmonicolor* JCM 21150^T^, sequence accession AJKI00000000 [102]
*Pedobacter agri* PB92^T^, sequence accession AJLG00000000 [103]
*Riemerella anatipestifer*, sequence accession CP003388 [104]
Phylum *Verrucomicrobia*
*Methylacidiphilum fumariolicum* strain SolV, sequence accession CAHT01000001 through CAHT01000109 [105]
Opitutaceae bacterium Strain TAV1, sequence accession AHKS00000000 [106]
